# Do speed amplitude and period affect gait variability and step followability under sinusoidal speed changing conditions?

**DOI:** 10.3389/fspor.2025.1602012

**Published:** 2025-06-02

**Authors:** Kiyotaka Motoyama, Takehiro Tashiro, Akira Saito, Masahiro Horiuchi, Taisuke Sakaki, Daijiro Abe

**Affiliations:** ^1^ Center for Health and Sports Science, Kyushu Sangyo University, Fukuoka, Japan; ^2^ Department of Information Design, Faculty of Design, Nishinippon Institute of Technology, Fukuoka, Japan; ^3^ Faculty of Sports and Life Science, National Institute of Fitness and Sports in Kanoya, Kagoshima, Japan; ^4^ Department of Mechanical Engineering, Faculty of Science and Engineering, Kyushu Sangyo University, Fukuoka, Japan

**Keywords:** gait stability, dynamic balance, planar covariation law, bipedal locomotion, kinematics, SPM

## Abstract

**Introduction:**

The time courses of the joint elevation angles of the thigh, shank, and foot in one stride during walking can be well approximated by a “*plane*” in a triaxial space. This intersegmental coordination (IC) of the lower limb elevation angles is associated with gait variability. This study aimed to examine how anteroposterior and lateral gait variabilities are influenced by different amplitudes (±0.33 vs. ± 0.67 m·s^−1^) and periods (30 vs. 60 s) of sinusoidal speed changes. We also examined which limbs are responsible for the step variabilities.

**Methods:**

The IC *plane* thickness and coefficient of variance of step width (CV_SW_) were quantified as anteroposterior and lateral gait variability in 18 young adults. Time delay of step length (TD_SL_) and step frequency (TD_SF_) against sinusoidal speed changes were determined. Two-way statistical parametric mapping was applied for the time courses of each limb angle.

**Results:**

The IC *plane* thickness was greater in the ±0.67 m·s^−1^ condition than the ±0.33 m·s^−1^ condition. Neither periods nor amplitudes affected CV_SW_, TD_SL_, and TD_SF_. In the middle gait cycle, shank and foot angles were delayed against sinusoidal speed changes in the ±0.67 m·s^−1^ condition during acceleration phase, whereas shank and thigh angles proceeded in that condition during deceleration phase.

**Conclusion:**

Amplitude of sinusoidal speed changes increased anteroposterior, but not lateral, gait variability regardless of period. Distal and proximal limbs are controlled differently when continuous step adjustments are required, and this may be attributed to step variabilities.

## 1 Introduction

The trajectory of the elevation angles of the thigh, shank, and foot in a gait cycle can be well approximated by a “*plane*” in a triaxial space (1), called the planar covariation law (PCL) (1–10). This approach contributes to showing the lower limb's spatiotemporal interlimb coordination (IC) during human gait. Moreover, the shape of the IC *plane* was altered by an abrupt perturbation of treadmill speed (3, 4). Thus, variability of the planarity of the IC *plane* in a gait cycle may be a result of the responses of individual lower limbs to maintain gait stability against the speed perturbation. Indeed, an increased degree of gait speed perturbations did not modify the IC *plane* planarity during compensatory behavior in the unperturbed leg (3), so that the IC *plane* planarity has been considered one of the inherent and robust kinematic universalities across several gait-related motor tasks (1, 3, 8). Some previous studies reported that gait speed modified the IC *plane* planarity (8–10), whereas others did not (5–7). This inconsistency may be derived from tested speed range. Studies without a speed dependency of the IC *plane* planarity measured narrower speed range from very slow to preferred walking speed (0.28–1.39 m·s^−1^) (5–7). Conversely, studies that found a speed dependency of the IC *plane* thickness did a relatively wider speed range (0.5–2.0 m·s^−1^) up to around gait transition speed (8–10). In addition, these previous studies tested the IC *plane* planarity at different steady-state gait speeds. Thus, more detailed study is necessary with regard to the impacts of measured speed range and speed changes on the IC *plane* planarity.

In our daily lives, passive gait speed changes necessarily occur based on changes in surface conditions (11) and visual illusion (12). A sinusoidal speed-changing protocol using a treadmill is particularly available to evaluate gait variability due to several reasons. First, it can involve a wide range of gait speed (13–15). Second, a consecutive spatiotemporal adjustment of the lower limbs is required for walkers without an abrupt perturbation (13–15). Third, the PCL concept can be established regardless of gait speed (1–10). Accordingly, we have recently examined the effects of sinusoidal periods of 30-, 60-, and 120 s with a ± 0.56 m·s^−1^ (±2 km·h^−1^) amplitude on gait variability (13). Although an abrupt speed change caused gait perturbation in association with a loss of the IC *plane* planarity (3, 4), our recent study did not observe such a great loss of the IC *plane* planarity when walking under sinusoidal speed changing conditions with different periods (13). This inconsistency may be derived from the rate of changing speed. Therefore, the magnitude (amplitude) of gait speed changes in a sinusoidal manner may play a key role in impairing the IC *plane* planarity. There is another benefit to employ sinusoidal speed changing protocol. We have found that a combination of stride length (SL) and step frequency (SF) was not necessarily optimized during walking (14, 15), even though the product of the step length (SL) and step frequency (SF) should correspond to the treadmill speed. These recent results suggested that the rate of changing speed might affect the lower limb adjustment during walking. The time delay (TD) of the SL (TD_SL_) and SF (TD_SF_) could reflect inappropriate adjustment of the lower limbs, resulting in possible step variabilities. Given these backgrounds, sinusoidal speed changing protocol can reveal how much SL or SF is delayed or proceeded when continuous step adjustment is required. Although a limited number of previous studies have examined the IC *plane* planarity under speed-changing conditions (3, 4, 13–15), the IC *plane* thickness varied with gait speed (8–10). Thus, these previous results provide a hypothesis that the greater the magnitude of sinusoidal gait speed change, the greater the variability of the IC *plane* planarity.

A stable gait with controlled multiple joints must be maintained by continuous adjustments of SL and SF, so that the time delay (TD) of SL (TD_SL_) and SF (TD_SF_) could reflect delayed adjustment of the lower limbs against sinusoidal speed change (14, 15). This is because step variabilities refer to the ability of the neuromuscular system to adapt to changing gait conditions (16, 17). In a greater speed amplitude condition, TD_SL_ and TD_SF_ in association with lateral gait variability evaluated by step width (SW) variability (18–23) would be greater because the neuromuscular system may not have sufficient time to achieve appropriate adjustment of the lower limbs at a greater amplitude of sinusoidal speed change. Accordingly, it was also hypothesized that the greater the speed amplitude, the larger the TD_SL_, TD_SF_, and SW variabilities. In addition, we further questioned which limb(s) are attributed to a followability of SL-SF combinations against sinusoidal speed change. This study aimed to examine the effects of amplitude (magnitude) and period of sinusoidal speed change on the variabilities of the IC *plane* planarity, SW variability, and followability of SL and SF.

## 2 Materials and methods

### 2.1 Participants

The G*Power 3.1 (24) was used to estimate the required number of participants with a statistical power of 0.8, a medium effect size (*f* value) of 0.25 proposed by Cohen (25), an alpha level of 0.05, and correlations among repeated measures of 0.8; at least 11 participants would be necessary. Under considerations of possible withdrawal due to students’ busy schedule, this study involved 18 healthy young adults. [7 men and 11 women; 20.7 ± 1.0 years old, mean ± standard deviation (SD)] without injuries. Their body height and mass were 1.649 ± 0.067 m and 60.9 ± 7.9 kg, respectively. An ethical committee established in Kyushu Sangyo University (No. 2019-0002 and 2024-0013) approved all procedures. Following the Declaration of Helsinki, all participants signed a written informed consent after being provided information about the purposes, experimental procedures, and possible risks of this study.

### 2.2 Procedure and data collection

**Figure 1:**
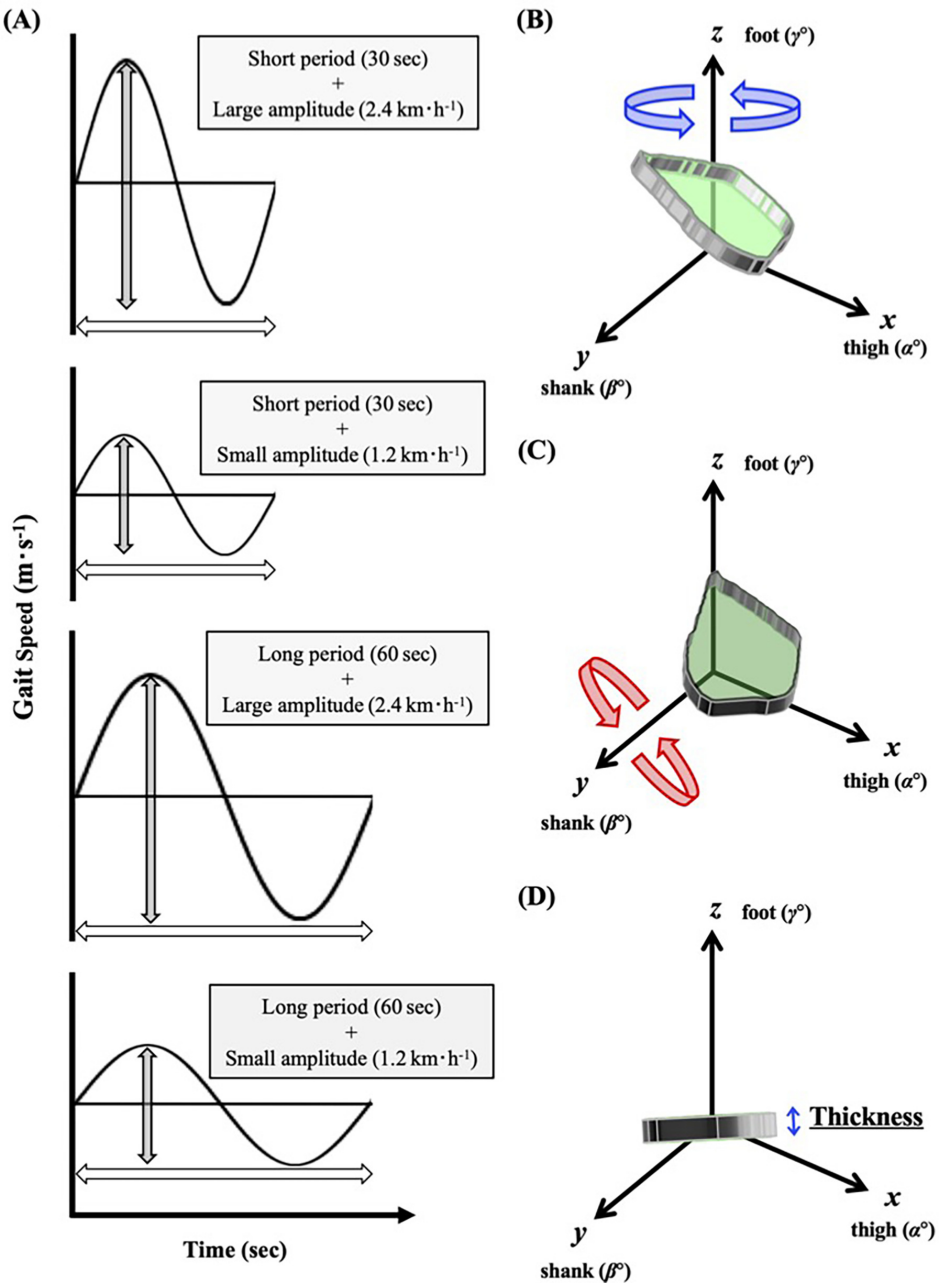
Protocols and eular's rotation of planar covariation *plane* to determine the thickness of interlimb coordination. **(A)** study protocols. **(B)** The best-fitting loop of the elevation angles of the thigh, shank, and foot is plotted in a squared *x*-*y*-*z* space as a "*plane*". **(C)** The best fitting "*plane*" is rotated around the *z* and *y* axes (shaded in green). **(D)** The z angle, at which the smallest standard deviation was obtained, is considered as the thickness of the spatiotemporal interlimb coordination.

We instructed the participants to put on compression shirts, half spats, and the same shoes in different sizes (ADIZERO-RC, Adidas Japan, Tokyo). The participants started walking on a motor-driven treadmill (TKK3080, Takei Scientific Instruments, Niigata, Japan) at 1.33 m·s^−1^ for males or 1.25 m·s^−1^ for females for 30 s as the baseline speed (i.e., midpoint speed during sinusoidal walking), followed by a preliminary habituation and warming-up walk. These baseline speeds were determined based on the metabolically economical walking speed, which was equivalent to the preferred walking speed observed in our previous studies (17, 26, 27). To determine the amplitude of sinusoidal speed changing protocol, we considered the fastest gait speed not to transit from walking to running (gait transition speed) (28). It almost appears around 2.0 m·s^−1^ (7.2 km·h^−1^) (28), so that the maximal amplitude was set ±0.67 m·s^−1^. To compare different amplitude of sinusoidal speed change, we also set ±0.33 m·s^−1^, which is just half of ±0.67 m·s^−1^. Subsequently, the treadmill speed was changed in a sinusoidal manner of 60- and 30 s periods with speed amplitudes of ±0.33 m·s^−1^ (±1.2 km·h^−1^) and ±0.67 m·s^−1^ (±2.4 km·h^−1^) in a randomized order (Figure 1A).

Based on recent studies (13, 14), twelve reflective markers were put on both lateral greater trochanters, shoulders (acromion), ankles (lateral malleolus), knees (lateral femur epicondyle), heels (backend of each shoe), and toes (toe of each shoe). Moreover, four additional markers were put on each corner of the treadmill. Motion data were captured using eight high-speed cameras (Kestrel300, MAC3D System, Rohnert Park, CA, USA) with a sampling rate of 100 Hz. The root mean square errors in calculating the three-dimensional (3D) marker locations were within 1.0 mm. The whole gait cycle, defined from the heel-contact to the toe-off, was divided into distinct parts in the range of 0%–100%. We computed the 3 × 3 matrix of the elevation angles of the lower limbs from the marker locations (Figure 1B) at each time frame. Furthermore, the best-fit 3D covariation loop (IC *plane*) did not perfectly lie on the *plane* (2, 5–10, 13, 14), and the IC *plane* seems to fluctuate during walking in a sinusoidal speed-changing condition (13, 14). Considerably large variations in the IC *plane* thickness could be observed if the shoe sole slightly rubbed the treadmill belt before the real heel strike. Thus, each sinusoidal cycle was continuously repeated thrice to avoid such incomplete motions. Even though the first sinusoidal period was fundamentally used for the subsequent analyses, the second or third cycle was used only when the shoe sole slightly hit the treadmill belt before the real heel strike in the earlier cycles. Accordingly, the largest standard deviation or mean value was not used to represent the IC *plane* thickness, which was considered the smallest standard deviation of the fluctuating IC *plane* in one gait cycle (Figure 1C).

In a practical computational calculation, the best-fit 3D approximation of the angular covariation is not a dimensionless *plane*. Therefore, based on the definition of Euler's angle, after detecting the best-fit IC *plane* of the 3D covariation was detected, it was rotated around the *z*-axis (foot elevation angle) as follows (13, 14):

(1) $$\left(\begin{array}{c} \alpha z\\ \beta z\\ \gamma z \end{array}\right)\;\;=\;\;\left(\begin{array}{ccc} \cos\;\theta z & \sin\;\theta z & 0\\ -\sin\;\theta z & \cos\;\theta z & 0\\ 0 & 0 & 1 \end{array}\right)\left(\begin{array}{c} \alpha \\ \beta \\ \gamma \end{array}\right)$$

where *α*, *β*, and *γ* are the original best-fit covariations, and *α*_z_, *β*_z_, and *γ*_z_ are the covariations after rotating around the *z*-axis. The matrix described by Equation (1) was simultaneously rotated around the *y*-axis (knee elevation angle) as follows:

(2) $$\left(\begin{array}{c} \alpha y\\ \beta y\\ \gamma y \end{array}\right)=\;\left(\begin{array}{ccc} \cos\;\theta y & 0 & \sin\;\theta y\\ 0 & 1 & 0\\ -\sin\;\theta y & 0 & \cos\;\theta y \end{array}\right)\left(\begin{array}{c} \alpha z\\ \beta z\\ \gamma z \end{array}\right)$$

where *α*_y_, *β*_y_, and *γ*_y_ are the covariations after rotating around the *y*-axis. Thus, the *plane* was rotated by a combination of the matrices 1 and 2 as follows:

(3) $$\left(\begin{array}{c} \alpha^{\prime }\\ \beta^{\prime }\\ \gamma^{\prime } \end{array}\right)=\left(\begin{array}{ccc} \cos\theta y & 0 & \sin\theta y\\ 0 & 1 & 0\\ -\sin\theta y & 0 & \cos\theta y \end{array}\right)\left(\begin{array}{ccc} \cos\theta z & \sin\theta z & 0\\ -\sin\theta z & \cos\theta z & 0\\ 0 & 0 & 1 \end{array}\right)\left(\begin{array}{c} \alpha \\ \beta \\ \gamma \end{array}\right)=\left(\begin{array}{ccc} \cos\theta y\;\cos\theta z & \cos\theta y\;\sin\theta z & \sin\theta y\\ -\sin\theta z & \cos\theta z & 0\\ -\sin\theta y\;\cos\theta z & -\sin\theta y\;\sin\theta z & \cos\theta y \end{array}\right)\left(\begin{array}{c} \alpha \\ \beta \\ \gamma \end{array}\right)\;$$

Considering both rotation angles, *θ*_z_ and *θ*_y_, ranging from 0° to 179°, 32,400 (180 × 180) combinations can be defined. Subsequently, the IC *plane* thickness was calculated in a non-arbitrary computational space.

The motion data were also used to calculate the TD_SL_ and TD_SF_ against sinusoidal speed change. The following equation was used to approximate SL and SF:

(4) SLand/orSF=Amp⋅sin(wt−TD)

where Amp, *ω*, and *t* represent amplitude, angular frequency, and time (ms), respectively. The SW was quantified as the lateral distance between both heel makers in each step (13, 14) because it was reported to be less dependent on the gait speed (18–23). Thus, the SW was measured during the whole first period (30 or 60 s) to calculate the coefficient of variance (CV_SW_; %) as the SW variability.

### 2.3 Statistical analysis

Data normality of measured gait parameters was assessed using Shapiro–Wilk test by GraphPad Prism (Ver.10 for MacOS, GraphPad Software, La Jolla, California, USA). After confirmed data normality, two-way (30 and 60 s periods × ± 0.33^−1^ and ±0.67 m·s^−1^ amplitudes) repeated measures analysis of variance (ANOVA) was used for comparisons of the dependent variables. To examine which limb(s) are attributed to TD_SL_ and/or TD_SF_, we applied two-way statistical parametric mapping (SPM) for the relative time series of each limb (29). The time series data were divided into the acceleration and deceleration phases. Statistical significance was set at *p* < 0.05. All data were presented as mean ± SD.

## 3 Results

**Figure 2:**
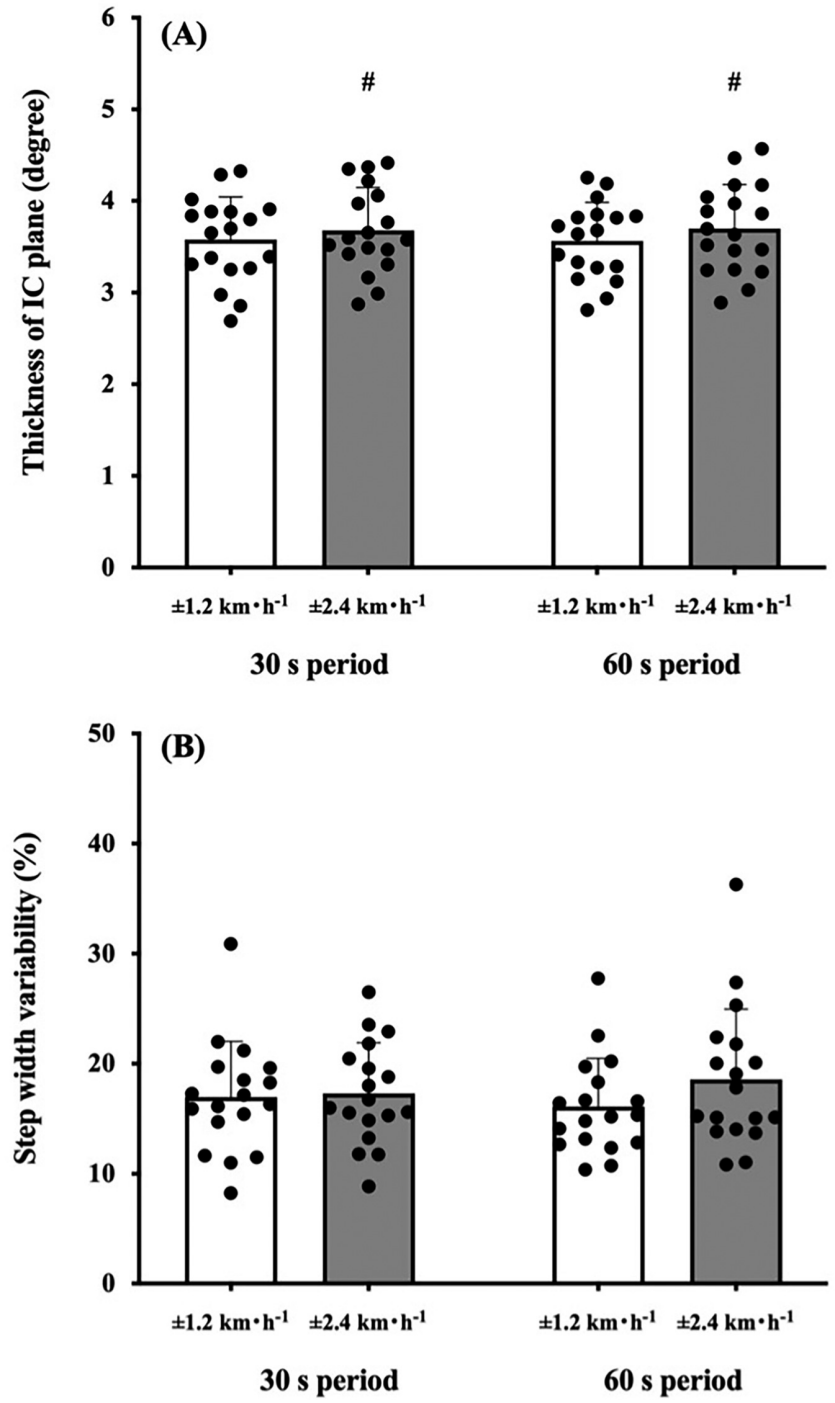
Comparisons of the interlimb coordination (IC) *plane* thickness and step width variability. **(A)** Participants walked on a treadmill with sinusoidal speed-changing protocols for time periods of 30 s and 60 s periods (left) with amplitudes of ±1.2 km·h^−1^ (white bars) and ±2.4 km·h^−1^ (dark bars), respectively. ±2.4 km·h^−1^ was significantly greater in the IC *plane* thickness. #*p* < 0.05. **(B)** The coefficient of variance values of the step width variability (CV_sw_; %) were compared between conditions and periods. The CV_sw_ was not significantly different between periods and conditions. Values are presented as means ± standard deviation.

**Figure 3:**
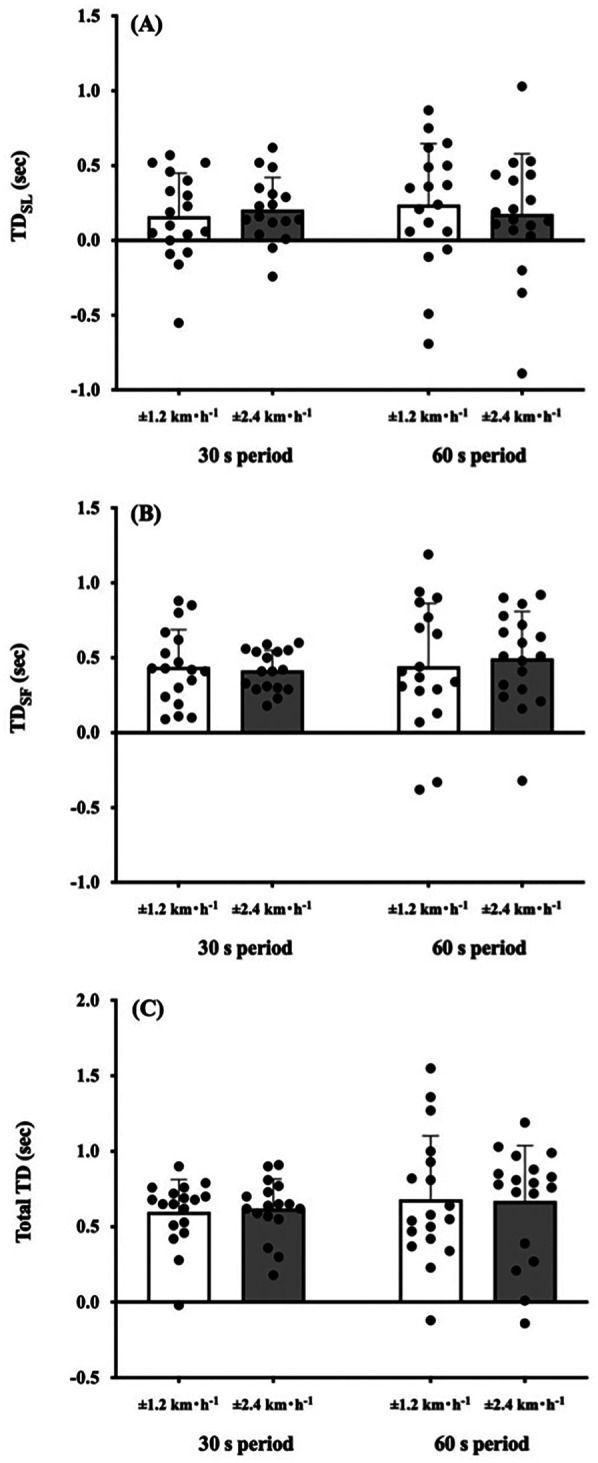
Comparison of time delay in step variabilities against sinusoidal speed change. **(A)** Time delay (TD) of step length (SL) against sinusoidal speed change. **(B)** TD of step frequency (SF). **(C)** Total TD. No significant differences were found between periods and amplitudes in these parameters. Values are presented as means ± standard deviation.

**Figure 4:**
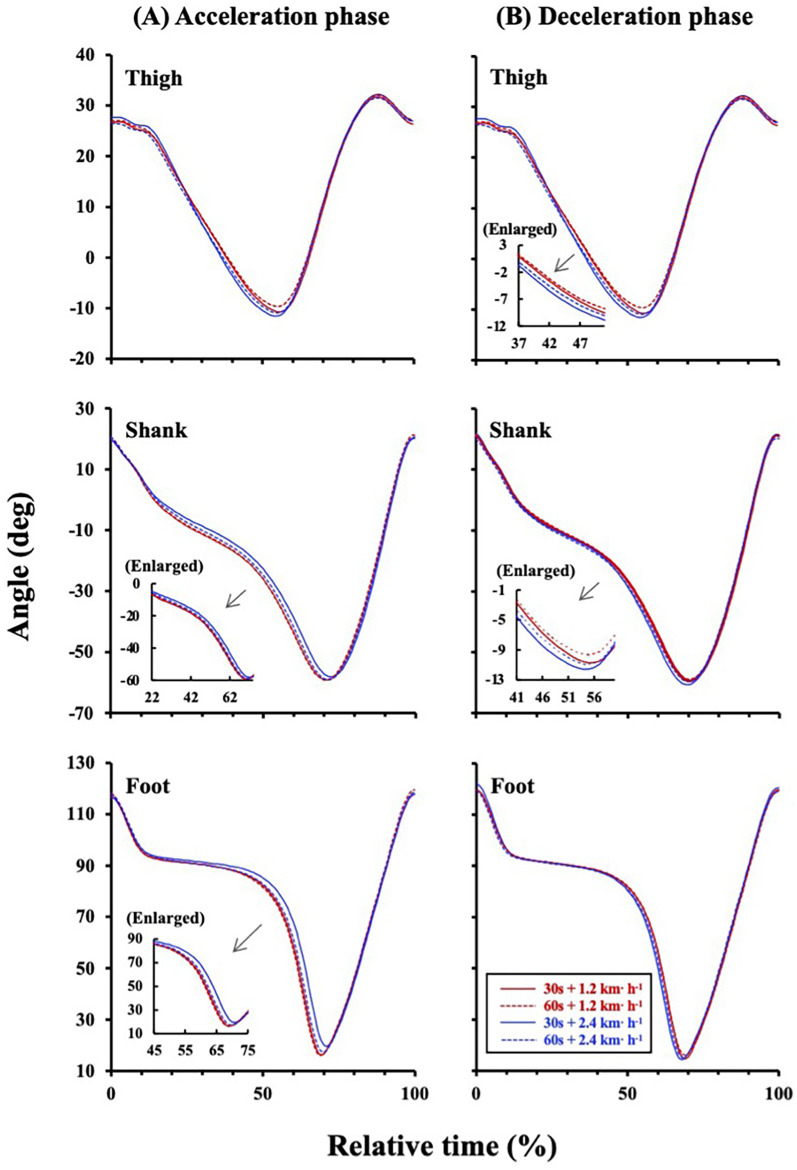
Relative time series of the lower limbs during acceleration and deceleration phases. **(A)** During acceleration phase, the shank and foot were significantly delayed in the ±0.67 m·s^−1^ condition (blue solid and broken lines) than in the ±0.33 m·s^−1^ condition (red solid and broken lines). Enlarged figures were inserted into the left middle panel (*p* < 0.05 at 22%-74%) and left bottom panel (*p* < 0.05 at 45%–75%). **(B)** During deceleration phase, the thigh and shank were significantly delayed in the ±0.67 m·s^−1^ condition (blue solid and broken lines) than in the ±0.33 m·s^−1^ condition (red solid and broken lines). Enlarged figures were inserted into the upper left (*p* < 0.05 at 37%–51%) and middle left panels (*p* < 0.05 at 41%–60%).

Two-way ANOVA showed a significant amplitude effect on the IC *plane* thickness (*F* = 10.286, *p* = 0.005; Figure 2A). A main effect of the sinusoidal period (*F* = 0.011, *p* = 0.919; Figure 2A) and interaction effect (*F* = 0.234, *p* = 0.635; Figure 2A) were not significant. The CV_SW_ trended to be greater in the ±0.67 m·s^−1^ condition than in the ±0.33 m·s^−1^ condition (*F* = 4.402, *p* = 0.051; Figure 2B), but this trend was not observed between the 30- and 60 s period (*F* = 0.083, *p* = 0.777; Figure 2B). The TD_SL_ was not significantly different between periods (*F* = 0.069, *p* = 0.796; Figure 3A) and amplitudes (*F* = 0.402, *p* = 0.534; Figure 3A). The TD_SF_ was the same as the TD_SL_ between periods (*F* = 0.012, *p* = 0.913; Figure 3B) and amplitudes (*F* = 0.657, *p* = 0.429; Figure 3B). Consequently, the total TD was not significantly different between periods (*F* = 0.090, *p* = 0.768; Figure 3C) and amplitudes (*F* = 0.222, *p* = 0.644; Figure 3C). At the middle gait cycle, the foot and shank angles were significantly delayed in the greater amplitude condition than in the smaller amplitude condition during the acceleration phase (Figure 4A), but the thigh and shank angles were significantly proceeded in the greater amplitude condition than in the smaller amplitude condition during the deceleration phase (Figure 4B).

## 4 Discussion

Most of the previous studies have examined the characteristics of the IC *plane* at several steady-state speeds (1, 2, 5–10, 16, 17) and demonstrated that gait speed influenced changes in the pattern of the intersegmental coordination of the lower limbs (2, 8–10, 16, 17). Our recent study revealed that different periods of sinusoidal speed change ranging from 30 s to 120 s did not modify the IC *plane* thickness in young active adults (13), indicating that anteroposterior gait variability is inherent in each individual. Based on these study backgrounds, we investigated how different amplitudes and periods of sinusoidal speed change influence gait variabilities and/or step variabilities in healthy young adults. In support of our first hypothesis, the greater the magnitude of the sinusoidal gait speed change, the greater the variability of the IC *plane* thickness (Figure 2A). The difference of ±0.67 m·s^−1^ and ±0.33 m·s^−1^ condition is the different rate of speed change. That is, the IC *plane* planarity was not necessarily robust if the rate of changing speed increased. Our present results were in line with some previous studies that the IC *plane* planarity was dependent on the gait speed (8–10). This could be due to a wider range of gait speed (0.67–2.0 m·s^−1^ for males and 0.59–1.92 m·s^−1^ for females) was used in our sinusoidal speed changing protocol. In the passive speed changing-condition, appropriate combinations of SL and SF were primarily important to follow the treadmill speed, indicating that efforts to avoid falls are expected to be integrated into step variabilities. Our present study showed that different periods and amplitudes of sinusoidal speed changing conditions did not influence TD_SL_ and TD_SF_ (Figures 3A,B), resulting in a non-significant difference in the total TD among the conditions (Figure 3C). In addition, the CV_sw_ was not significantly different among the conditions (Figure 2B), indicating that our second hypothesis that the greater the speed amplitude, the larger the TD_SL_, TD_SF_, and SW variabilities was rejected. Previous studies reported that there was a TD between thigh and shank motions even in young adults (9, 10). Such a TD in the shank-foot coordination may provide greater distortion of the IC *plane* planarity. Some considerations were still necessary because step variabilities are quite large because the coefficient of variance of the total TD was 97.6% (±0.33 m·s^−1^) and 53.3% (±0.67 m·s^−1^) at the 60 s period condition (Figure 3C), whereas relatively smaller CV_sw_ was found in the ±0.33 m·s^−1^ condition (26.5%) and ±0.67 m·s^−1^ condition (33.5%) at the 60s period condition (Figure 2B). Notably, excessive gait variability could be associated with increased fall risks not only in the elderly population (19–23) but also in young adults (30, 31); however, these large variations in the step variabilities may reflect flexible locomotor control ability against passive gait speed changes in healthy young adults.

Step variabilities are also associated with an ability of the neuromuscular system to adapt to changing gait conditions (16, 17), so that step variabilities could result in different time series of each limb. Thus, we compared the relative time series of each limb elevation angle to examine which limbs are attributed to TD_SL_ and/or TD_SF_. The TDs of the thigh-shank and shank-foot decreased as gait speed increased (8, 9), indicating that followability of the lower extremities was enhanced against treadmill speed particularly at faster gait speed. Indeed, we also observed that the shank and foot elevation angles were significantly delayed in the ±0.67 m·s^−1^ condition than in the ±0.33 m·s^−1^ at the middle gait cycle during the acceleration phase (Figure 4A). Conversely, the thigh and shank elevation angles significantly proceeded in the ±0.67 m·s^−1^ condition than in the ±0.33 m·s^−1^ condition during the deceleration phase (Figure 4B). That is, more distal limbs were delayed in greater amplitude conditions than in the smaller amplitude conditions at the middle gait cycle during acceleration phase, whereas more proximal limbs proceeded in these conditions during the deceleration phase. These opposite behaviors between the distal and proximal limbs during acceleration and deceleration phases can induce small perturbations that would generate torque to push or pull the center of body mass (COM). Indeed, ground reaction force passes in front of the COM at the heel strike, and it does behind the COM at the toe-off (32, 33), which is a little-known biological feature only in human bipedal walking. Such a generated torque can restore dynamic gait stability similar to a “passive walk” performed by a bipedal robot that intentionally creates an unstable state with a perturbation (33–36). A passive walk is characterized by lesser energy cost (37), and this may be related to a high efficiency of human gait (38, 39). Since the thigh and shank angles are controlled by hip and knee joints, the knee flexion determined by these angles could play an important role in allowing toe clearance during the swing phase and in facilitating shock absorption during the stance phase. In a sinusoidal speed changing condition requiring continuous step adjustments, relatively greater TD_SL_ and TD_SF_ still existed even in young adults only (Figures 3A, 3B), which was in line with our recent studies (14, 15). These results suggested that anatomical functions of shock absorption and allowing toe clearance should not adequately activate even if a quick and proper adjustment of SL and SF is necessary. Different distal and proximal limbs controls (Figures 4A,B) would be necessary to compensate such situations.

In addition to the above-mentioned passive walker model, leg joint stiffness in humans is different among each joint, and those joint stiffness alters during the gait cycle (40), particularly in the foot. This is due to three arches of the foot to absorb passive reaction forces from the ground. This anatomical function of the foot would be one of the sources explaining the delayed time course of the foot elevation angle during the acceleration phase (Figure 4A) as stated before. The knee joint also has an interesting feature of increasing stiffness during the stance phase and decreasing it during the swing phase (40). A decrease in the knee joint stiffness during the swing phase is controlled by releasing the co-contraction of thigh antagonist muscles to facilitate lower leg movement during the swing phase, which can potentially contribute to minimize energy costs of walking. Such a dynamic change in the knee joint stiffness have also been observed during hopping (41, 42). Taken together, the proximal thigh is primarily controlled quickly, instead, the distal shank and foot were delayed at the middle gait cycle during the acceleration phase in response to sinusoidal speed changes (Figure 4A). On the contrary, the proximal thigh takes precedence against sinusoidal speed change during the deceleration phase to create a time margin for executing successful next step adjustment (Figure 4B). Consequently, highly accurate step-by-step control in response to the passive force from the ground must be given up for adopting a strategy of gait stability within a few steps.

A sinusoidal speed changing condition is almost equivalent to a gradual speed changing condition. As demonstrated (12), visual illusion makes walkers change their preferred gait speed, which is likely to occur at dusk. A gradual gait speed change in association with a passive step adjustment should necessarily occur at a pedestrian crossing or at a railroad crossing just before a train passes. It should also occur if slippery and non-slippery surfaces are mixed on icy roads or in busy places. Thus, we are being forced to face with gradual speed changes in our daily lives without realizing it. However, a study limitation should be stated because our present study involved only young adult participants. Since this study was originally aimed to explore potential factors that cause gait and step variabilities in human bipedal walking, it could be rather appropriate to limit to young participants in order to exclude the age effect. Therefore, the interpretations of our present results should not be easily expanded to aged populations or clinical patients.

## 5 Conclusions

Greater amplitude of sinusoidal speed change increased anteroposterior gait variability, but not lateral gait variability, regardless of periods even in healthy young adults. The time courses of more distal limb elevation angles were delayed in greater speed amplitude conditions during the acceleration phase, whereas the time courses of more proximal limb elevation angles proceeded in that condition during the deceleration phase. These different behaviors of the lower limb segments suggest that the distal and proximal limbs are controlled differently when continuous step adjustment is required during walking, and this may be attributed to step variabilities.

## Data Availability

The original contributions presented in the study are included in the article/Supplementary Material, further inquiries can be directed to the corresponding author.
